# Development and Evaluation of an Online Education-Entertainment Intervention to Increase Knowledge of HIV and Uptake of HIV Testing among Colombian Men Who Have Sex with Men (MSM)

**DOI:** 10.3390/ijerph18041811

**Published:** 2021-02-12

**Authors:** Ana María del Río-González, Maria Cecilia Zea, Sarah K. Calabrese, Fabián Betancourt, Jorge Pacheco-Cabrales, Yacid Estrada-Santiago, Paul J. Poppen

**Affiliations:** 1 Department of Psychological and Brain Sciences, The George Washington University, Washington, DC 20052, USA; zea@gwu.edu (M.C.Z.); skcalabrese@gwu.edu (S.K.C.); pjp@gwu.edu (P.J.P.); 2 Liga Colombiana de Lucha Contra el SIDA (LigaSida), Bogotá 111311, Colombia; fabian.betancourt@gmail.com (F.B.); jpcabrales@yahoo.com (J.P.-C.); yacidestrada@gmail.com (Y.E.-S.)

**Keywords:** promotion of HIV testing, men who have sex with men (MSM), intentions to test, knowledge of HIV

## Abstract

Using a community-based participatory approach, we developed a film to promote HIV testing among young men who have sex with men (MSM) in Bogotá. Using a 5-step process to develop the intervention, we conducted 11 focus groups with MSM (n = 141) to receive community feedback at each step. To evaluate the intervention we recruited 300 young MSM to complete a baseline survey in December 2017. Between February–June 2018, 63 participants watched the film and completed a post-viewing survey, which showed the intervention was acceptable for the target population. Between August–December 2018, 48 MSM who watched the film and 47 who did not (control group) completed a follow-up survey. To obtain preliminary evidence of the efficacy of the intervention, we assessed the main effect of time (baseline vs. follow-up) and the interaction between time and group (intervention vs. control) on HIV testing uptake and intentions, and knowledge of HIV transmission dynamics and HIV-related rights. Knowledge of HIV rights increased from baseline to follow-up in the intervention group only. HIV Knowledge increased for both groups. HIV testing intentions increased significantly more for non-gay-identified men in the intervention group, but the overall effect of the intervention was not significant. Testing uptake did not change.

## 1. Introduction

As in many low- and middle-income countries, Colombian cisgender men who have sex with men (MSM) constitute a group at high risk for HIV, with a prevalence of 17% for this group in 2011 [1]. In Bogotá, prevalence of HIV ranged from 12% to 15% in two large samples of MSM [1,2]. Awareness of HIV status is critical for halting the epidemic, but HIV testing among Colombian MSM is low. We conducted a survey in Bogotá in 2011-2012 and found that 50% of MSM had never tested for HIV [3]. The situation is more dire in other Colombian cities, where estimates of untested MSM ranged from 59% to 86% [1]. Increased detection and treatment of HIV are crucial goals, because these actions can reduce new transmissions and enable earlier treatment and thereby better health outcomes [4,5,6].

Telenovelas, which are time-limited melodramatic soap operas, are extremely popular in Latin America, and previous research has shown that they afford a culturally congruent and effective means of providing health information and promoting health behaviors [7,8,9,10,11]. Telenovelas can be powerful vehicles affecting social norms, attitudes, and health behavior through a communication strategy called education-entertainment [12,13,14,15,16]. For example, education-entertainment programs have been shown to lessen homonegativity in Mexico [17], increase intentions to test for syphilis among MSM in the U.S. [9], and change knowledge and attitudes toward breast cancer screening among Latinas in the U.S. [10]. Although telenovelas are typically seen as television programs, the format is well suited to web-based postings, and the “webnovela” has been touted as an important future direction in the field of education-entertainment [18].

The use of internet technologies to deliver HIV prevention interventions targeting MSM has increased steadily since 2007, but few are video-based. In a 2019 systematic review of 55 eHealth HIV/STI prevention interventions for MSM, Nguyen and colleagues found that only seven were video-based [19]. Of these video-based interventions, three were conducted in China [20,21,22], three in the United States [23,24,25] and one in Peru [26]. Thus, knowledge about the feasibility and acceptability of using this type of intervention among MSM in Latin America is very limited. The study conducted by Blas and colleagues in Peru [26] found that a 5-min HIV testing motivational video was effective to increase testing intentions and behaviors, but only among non-gay-identified MSM, showing that sexual orientation might moderate the effect of these interventions.

In this article, we describe the development and evaluation of an education-entertainment online video intervention to increase knowledge of HIV and uptake of HIV testing among young Colombian MSM. In this paper’s introduction, we describe how we used community-based participatory research (CBPR) to develop the video intervention. In the methods and results sections, we present the evaluation of acceptability and preliminary effectiveness of the video intervention.

### Development of an Education-Entertainment Online Video Intervention Using CBPR

Community-based participatory research is a valuable way to empower people and groups, and enhance their voices [27]. As an orientation to research, CBPR strives to address health needs from an ecological perspective and it is grounded on the strengths and resources of the community, equalizes power among participants and researchers, and integrates knowledge acquisition and interventions for the mutual benefit of all partners [28]. We formed a CBPR team composed of: (1) Young MSM (ages 18 to 30) from Bogotá, Colombia; (2) Members of community organizations serving MSM; (3) Experts in entertainment-education and media production; and (4) Researchers and research staff.

We used a 5-step process, consistent with methods for the development of education-entertainment interventions [29]. These processes include first, developing an evidence-based message to promote health behavior change in the target community. Second, to convey to media development professionals the communication intentions of the intervention (Message Brief). Third, to develop the story. Fourth, to write the script. Fifth, to produce and edit the film. To receive community feedback at each step, we conducted 11 focus groups with young MSM who were not part of the CBPR team (n = 141). We also interviewed five key informants working in relevant areas (e.g., Infectious disease physicians, HIV prevention specialists from the Department of Health) to ensure an evidence-based approach was consistently used. Table 1 presents the major activities, specific objectives, significant results and key outcomes for the five steps.

Step 1: Background research on the target population, the message topic, and the communication environment. Information gathered in our previous study on MSM in Bogotá provided the initial foundation of the background research for this intervention. We conducted additional formative research to determine the appropriate objectives, messages, media, formats, and images to achieve the intervention goals with the specific target audience. The CBPR team met to explore characteristics, circumstances, and behavior of the target population of young MSM in Bogotá, and to examine the individual, social, and structural conditions facilitating and impeding HIV testing. Additional topics discussed by the CBPR team included the HIV epidemic in Colombia, transmission dynamics, and public health strategies such as test-and-treat approaches. In addition, the CBPR team discussed the need for the intervention to be sex-positive and affirming of diverse sexual orientations and gender identities, within a framework of human rights and gender equality.

**Figure 1:**
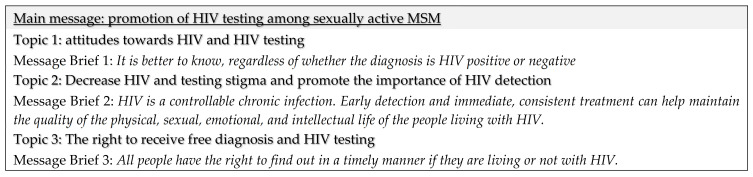
Message Brief.

Step 2: Development of the Message Brief. The CBPR team also met to develop the Message Brief, the document describing the crucial messages to bring about the desired changes in the target community, and the research supporting those messages. The primary message focused on the importance of regular HIV testing among sexually active MSM. In addition, other messages supporting the primary one included (1) early detection and treatment of HIV results in better health outcomes of infected individuals; (2) HIV-infected individuals can live full, productive lives; and (3) public policy in Colombia establishes a right to up to two HIV tests per year. To assess the clarity and suitability of these messages, we conducted two focus groups with young MSM in Bogotá (n = 15) and interviewed the five key informants. Based on their feedback, the CBPR team revised the final Message Brief (see Figure 1).

Step 3: Development of broad story outline and characters. The CBPR team and the creative team of script-writers discussed possible characters, setting, plot, and actions. Then the creative team wrote a draft story outline, integrating the messages into a drama, with the aim of achieving the appropriate balance between entertainment and education. We aimed to engage the viewer and elicit emotional involvement and empathetic responses, while conveying the key messages. To achieve this aim, the story portrayed role model characters who became empowered in the course of the story and whose behavior had the potential to influence their community and their own lives. The story portrayed specific challenges. For instance, characters who avoided HIV testing did so for a variety of reasons, such as fear of being positive, fear of a breach of confidentiality, low risk perception, and lack of awareness of the right to free testing. To obtain community and expert feedback we conducted three focus groups with young MSM (n = 30) and five key informant interviews. They appraised the story, characters, and messages in the draft story outline. The CBPR team examined all of the feedback and determined aspects that needed further changes that the creative team used to revise the outline.

Step 4: Development of the script. The creative team wrote detailed story lines, character sketches, scripts, and scenes that the CBPR team examined and refined prior to pre-testing. Pre-testing of the scripts examined the quality of both the key messages and the entertainment component. We used an iterative process to evaluate and revise the scripts. We conducted three focus groups (n = 51) to obtain feedback from members of the target community of MSM. We addressed three main questions: (1) Are the story and the characters compelling, relevant, credible, and culturally appropriate? (2) Are the intended messages clear? (3) Are there unintended messages conveyed? We conducted interviews with five key informants who reviewed the scripts for accuracy of the informational content, as well as reactions to the story content, the salience and clarity of the messages, and the balance between the entertainment and educational aspects. Based on these qualitative data the CBPR team identified areas in need of modification and made recommendations to the creative team to revise the story lines, characters, and scripts.

**Figure 2:**
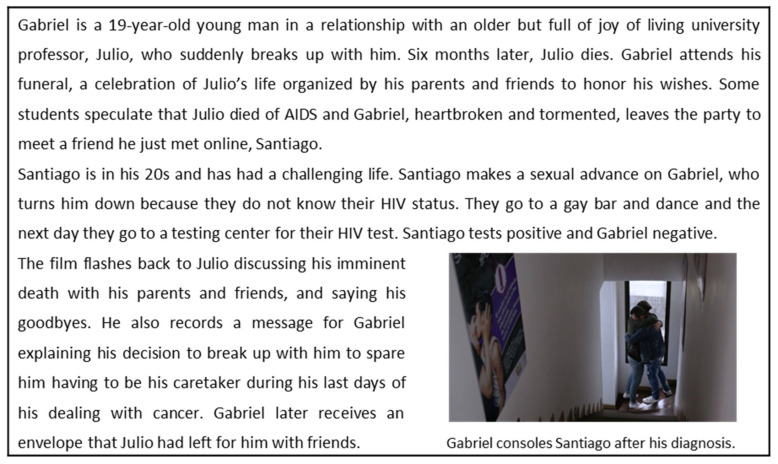
Film synopsis.

Step 5: Production. We hired a producer who was an expert in educational entertainment and film production, a director, and an assistant director to produce the webnovela. Three focus groups of young MSM (n = 40) provided feedback on the actors selected for casting and the final scripts. The majority of the actors, including the main characters, were natural actors, members of the MSM and the transgender communities in Bogotá. We selected two members of the CBPR team to act as “message guardians” who were at the production set to ensure that decisions made during filming did not obscure the primary and secondary messages. The CBPR team was apprised of progress and was available to address any questions or issues during production. After shooting, the director and a film editor worked on the final product. After editing, the director and the producer considered that the original five episodes format was not ideal given some of the artistic decisions the director made during filming. One of such decisions was the shooting of a sequence plane, a plane that registers an action in an entire sequence, without any cuts, which was great cinematography but it made the story slow and possibly not engaging enough for the viewers. Thus, the final product was an 83-min feature film. The CBPR team voted for “*Bondage: It is better to know*” as title for the film. Figure 2 presents the synopsis of the film.

Our primary target audience for the intervention included MSM who were HIV-negative or who did not know their HIV status, because of the public health goal of the study was to increase diagnosis in this group. A secondary audience included MSM living with HIV, who constitute a group that can potentially influence the behavior of the primary target audience. MSM living with HIV could encourage other MSM to be tested, dispel misconceptions about HIV, and serve as models living successfully with the infection.

In the following sections, we describe the results of the evaluation of “*Bondage: It is better to know*” as an online educational entertainment video intervention to promote HIV testing among young adult MSM in Colombia. Specifically, we assessed acceptability of the film and explored its effects on HIV testing intentions and behavior, and knowledge of HIV transmission dynamics and HIV-related rights.

## 2. Materials and Methods

### 2.1. Study Design

We conducted a feasibility and acceptability pilot randomized controlled trial of “*Bondage: It is better to know”*. All procedures were approved by the George Washington University IRB and the ethics committee from La Liga Colombiana de Lucha contra el SIDA.

We administered an in-person baseline survey in December of 2017 (N = 300). We used respondent-driven sampling (RDS), a long chain-referral sampling strategy to recruit participants. RDS is an efficient, appropriate, and cost-effective method for obtaining hidden populations such as MSM [30,31]. RDS is theorized to produce a more representative sample than convenience sampling strategies and it provides estimates of population prevalence and information concerning the diversity and clustering within the sample [32]. For the present study, we used RDS for recruitment purposes only. RDS involves the following steps: A number of participants known to be members of the hidden population were recruited into the study. These participants act as seeds, who recruit others to participate and receive a monetary reward for their own participation, as well as the participation of each person they recruit. The participants recruited by the seeds then also act as recruiters and are rewarded for their own participation and for each participant they refer to the study. Additional waves of participants are recruited using this approach until the desired sample size is obtained. A recruitment quota for each participant is used to foster a broader selection of subjects and to limit the influence of any particular individual on the composition of the sample. Because of the incentives offered, precautions are necessary to protect the integrity of the sample. Referred individuals are carefully screened to establish whether they actually possess the defining trait of the population and to ensure that people do not participate more than once.

Our partner NGO in Colombia, LigaSida, identified and recruited three participants from diverse socioeconomic characteristics and living in different geographic regions of Bogotá to be the seeds to initiate referrals. Participants could refer a maximum of three MSM whom they personally knew using a coupon with a unique serial number to each potential recruit. Interested candidates would contact the project, which protects the privacy of members of stigmatized groups, as the identity of potential recruits is not known to the researchers unless the individuals choose to initiate contact. Referrals who contacted the study received additional information about the study, underwent screening for eligibility, and made an appointment for participation. Participants were told that the study was investigating the use of web-based materials to provide information about HIV.

**Figure 3:**
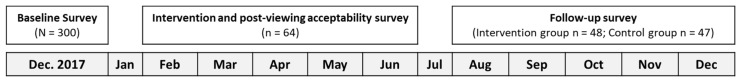
Timeline of the evaluation of *“Bondage: It is better to know”*.

Following our original plan, we randomly assigned baseline participants to the intervention and the control group (n = 150 each). In February 2018, we emailed unknown HIV status and HIV-negative participants in the intervention group (n = 135) a link to view the film online and respond to a post-viewing acceptability survey. Because only 31 participants from the intervention group had completed the post-viewing survey by April 2018, we invited an additional 50 participants from the control group to complete this phase. In total, 97 (52.4%) participants accessed the post-viewing survey by the end of June 2018 but only 63 (34.1%) responded to it. Six months after completing the post-viewing survey, the 63 participants who viewed the film were invited to complete an in-person follow-up survey, and 48 (76.2%) completed the follow-up survey. Participants who had not watched the video were also invited to complete this in-person survey during this period, serving as the control group for this phase. Forty-seven (52.2%) participated in this phase. Figure 3 describes the timeline of the evaluation of “*Bondage: It is better to know*.” Participants were reimbursed for their time in Colombian pesos $65,000 (USD $22) for the baseline survey and $75,000 (USD $25) for the follow-up survey. In addition, we paid $20,000 Colombian pesos (USD $7) for each participant they recruited. We did not reimburse participants in the intervention group for the post-viewing acceptability survey.

### 2.2. Participants

Inclusion criteria for participation were: self-identifying as MSM, being 18 to 30 years of age, and living in Bogotá. HIV status was not an inclusion criterion for participation in the study, but we asked participants for their self-reported HIV status. We retained eligible participants who were living with HIV to gain their perspectives on the film and to maintain the integrity of the RDS chains. However, for the evaluation of the intervention presented in this paper, we focus on those participants who self-reported being HIV-negative or were unsure of their HIV status. Table 2 presents baseline demographic information for participants at all time-points.

### 2.3. Measures

The project included several measures for the baseline, post-viewing and follow-up surveys. Analyses in this paper focus on variables related to acceptability and preliminary efficacy of the education-entertainment intervention.

#### 2.3.1. Acceptability Measures

We assessed whether participants liked the video (1 = Not at all; 5 = A lot), found the video informative (1 = Not at all; 5 = A lot), would recommend the video to their friends (1 = No; 2 = Maybe; 3 = Yes) and thought their MSM friends would like it (1 = Not at all; 5 = A lot). We also assessed narrative engagement, counterarguing (i.e., “the generation of thoughts that explicitly refute a message’s intended persuasive theme” ([33] p. 758), and identification with the main character of the story (Gabriel), using or adapting items from scales validated in Spanish [34]. Items measuring narrative engagement (n = 10) were assessed using a five point scale (1 = Strongly disagree; 5 = Strongly agree). Response options for items measuring counterarguing (n = 4) and identification with the main character (n = 6) ranged from 1 (Not at all) to 5 (A lot). These measures were included in the post-viewing acceptability survey (Table 3; Spanish version in Supplementary Materials Table S2).

#### 2.3.2. Efficacy Measures

We assessed lifetime and last 12 months HIV testing history in baseline, last 3 months for post-viewing acceptability survey and last 6-months for the follow-up survey. At all time-points we also assessed HIV testing intentions (two items; 1 = Strongly disagree; 5 = Strongly agree), knowledge about HIV transmission dynamics (five items), and knowledge about HIV-related rights (four items). Table 4 presents descriptive results for these measures and includes the exact wording of the items (Spanish version of the items in Supplementary Materials Table S2).

### 2.4. Data Analysis

In addition to the descriptive analyses presented in Table 2, Table 3, Table 4, we present descriptive information for HIV testing behaviors and intentions, knowledge of HIV transmission dynamics and knowledge of HIV-related rights by time (baseline vs. Follow-up) and intervention group (intervention vs. control; Table 5). To assess preliminary efficacy of the intervention we used a repeated measures logistic regression model for recent HIV testing and repeated measures linear regression models for HIV intentions and knowledge. Regression models included group, time and the interaction between group and time, and controlled for age, sexual orientation (1 = Gay; 0 = Other), SES and education. Gender identity was not included as a control variable because all follow-up survey participants in the intervention group were cisgender men and only three participants in the control group were transgender (2 women, 1 men). Rather, we conducted two separate sets of regression analysis, one excluding and one including transgender participants from the control group. Both sets of analyses yielded similar findings. Values presented in the results are from the models with all participants.

We also conducted secondary analyses to explore whether the main effect of intervention group on each of our outcomes was moderated by sexual orientation or lifetime HIV testing history at baseline. Given the small sample size of some of the resulting groups (e.g., 10 participants in the intervention group that had never been tested at baseline), we used a non-parametric approach for these analyses. For analyses with each moderator, we created four groups defined by the interaction of group (i.e., intervention vs. control) and two levels of the moderator (i.e., sexual orientation: gay vs. other; lifetime HIV testing history at baseline: ever tested vs never tested). We used Pearson Chi-Square tests for our dichotomous outcome (recent HIV testing at 6-month follow-up) and Kruskal-Wallis tests for our continuous outcomes (difference between baseline and follow-up scores on HIV testing intentions, knowledge of HIV transmission dynamics and knowledge of HIV-related rights).

## 3. Results

### 3.1. Characteristics of the Baseline Sample (N = 300)

At baseline (N = 300) most participants reported having ever been tested for HIV (74.7%; n = 224). We asked about HIV status and 25 participants reported being HIV positive (8.3%), 187 HIV negative (62.3%) and 88 did not know their status (29.3%).

### 3.2. Randomization and Attrition Analyses

We initially randomized participants who reported being HIV negative or not knowing their HIV status into an intervention (n = 135) and a control group (n = 140). Due to low participation in the intervention group, we randomly selected 50 additional participants from the control group and moved them to the intervention group. Compared to participants in the control group, participants invited to the intervention group were more likely to identify as cisgender men (χ^2^ = 8.39, *p* = 0.015), and had higher knowledge of HIV transmission dynamics (t(273) = 2.39; *p* = 0.014) and HIV-related rights (t(273) = 2.03; *p* = 0.044). There were no significant differences between groups in terms of age, sexual orientation, SES, education, lifetime history of HIV testing or testing intentions.

Participants who accepted our invitation to participate in the intervention and who completed the post-viewing acceptability survey (n = 63), had higher knowledge of HIV transmission dynamics at baseline (t(175) = 2.39; *p* = 0.014) than intervention group participants who did not participate. Differences in baseline demographic characteristics, lifetime history of HIV testing, testing intentions and knowledge of HIV-related rights were not significant. Among post-viewing acceptability survey participants (n = 63), the only significant difference between those who completed the follow-up survey (n = 48) and those who did not (n = 15) was SES. Specifically, medium-SES participants were less likely to participate in the follow-up survey than lower-SES participants (χ^2^ = 5.22, *p* = 0.022). Among participants in the control group, the only difference between those who participated in the follow-up survey and those who did not was sexual orientation. Specifically, participants who identified as gay were less likely to participate in the follow-up survey than participants who identified as bisexual or heterosexual (χ^2^ = 9.41, *p* = 0.024).

### 3.3. Acceptability of the Intervention (n = 63)

Overall, reactions to the video were positive. As reported in Table 3, participants found the video informative (M = 4.21, SD = 0.94), liked the video (M = 3.79, SD = 1.02) and thought their MSM friends would like it too (M = 3.81, SD = 1.13). Furthermore, only five participants reported that they would not recommend the video to their friends. Narrative engagement was moderately high (M = 3.47, SD = 0.73) and counterarguing was low (M = 2.62, SD = 0.96). Identification with the main character (Gabriel) was moderately high (M = 3.43, SD = 1.10).

### 3.4. Preliminary Efficacy of the Intervention (n = 95)

#### 3.4.1. Recent HIV Testing

As presented in Table 5, there was a slight increase in recent testing from baseline to follow-up for both intervention (baseline = 47.9%; follow-up = 56.3%) and control groups (baseline = 42.6%; follow-up = 46.8%). Results from the repeated measures logistic regression, however, show that neither the main effects of group (B = 0.014; *p* = 0.97), time (B = 0.001; *p* = 0.99) or their interaction were statistically significant (B = 0.347; *p* = 0.54). These results suggest the intervention did not influence HIV-testing behaviors.

#### 3.4.2. HIV-Testing Intentions

HIV-testing intentions were high for both groups at baseline and follow-up (Table 5). The main effect of group (B = 0.114; *p* = 0.52), time (B = 0.161; *p* = 0.29) or their interaction were not statistically significant (B = 0.150; *p* = 0.46), suggesting that the intervention did not influence HIV-testing intentions.

#### 3.4.3. HIV Knowledge

Knowledge about HIV transmission dynamics increased significantly from baseline to follow-up for both groups. While the main effect of time was significant (B = 0.521; *p* = 0.003), the main effect of group (B = −177; *p* = 0.51) and the interaction between group and time-point were not significant (B = 0.206; *p* = 0.45). Similar findings were obtained for knowledge about HIV-related rights. Scores on this variable increased significantly from baseline to follow-up only for participants in the intervention group. However, while the main effect of time (B = 0.396; *p* = 0.019) was significant, neither the main effect of group (B = −0.116; *p* = 0.528) nor the interaction effect (B = −0.214; *p* = 0.385) were significant. These findings suggest the intervention was not effective in influencing HIV knowledge.

#### 3.4.4. Exploratory Moderation Analyses

Sexual orientation moderated the effect of the intervention on testing intentions (H = 8.63, df = 3, *p* = 0.035), such that intentions increased significantly more among non-gay-identified men in the intervention group than among gay-identified men on either the intervention or the control group. There were no moderation effects of sexual orientation on recent testing and HIV knowledge of transmission dynamics or rights.

History of HIV testing moderated the effect of the intervention on knowledge of HIV-related rights, (H = 8.08, df = 3, *p* = 0.044), such that knowledge of testing rights increased significantly more for men in the intervention group who had never been tested at baseline compared with men in all the other groups. There were no moderation effects of testing history at baseline on recent HIV testing, intentions to get tested, and knowledge of transmission dynamics.

## 4. Discussion

We aimed to examine the feasibility and acceptability of an online educational entertainment intervention to promote HIV testing among young adult MSM in Colombia. Our second aim was to assess the effects of the film on HIV testing intentions and behavior, and knowledge of HIV transmission dynamics and HIV-related rights, and to explore whether these effects were moderated by sexual orientation or HIV testing history.

The process of developing the intervention was grounded in community-based participatory research and this collaboration was very productive. The CBPR team was composed of young MSM, members of community organizations serving gay men, experts in entertainment-education and media production, and the researchers. At the time we developed the intervention, the need to promote HIV testing among young MSM in Colombia was critical. Based on a national survey data in Colombia, as few as one in nine individuals living with HIV were aware of their positive serostatus [35]. Moreover, despite the fact that MSM constitute a group at high risk for HIV, only 14% to 41% of MSM in seven Colombian cities reported ever having been tested [1]. In our previous research, we found that half of MSM sampled in Bogotá had never had an HIV test [3]. Current policies promoting test-and-treat approaches are based on the finding that increased diagnosis, treatment, and adherence result in greater viral suppression, and consequently, decreased HIV transmission and incidence [6]. Early detection and treatment of HIV is also associated with better health outcomes for individuals living with HIV [36,37,38,39]. For these reasons, developing effective strategies to increase HIV testing is a high priority research topic. In Colombia, semi-annual free HIV testing is mandated as a right [40]; however, policies and bureaucratic regulations in the health-care industry often contravene this rule [3]. Despite this right, we found that 60% of HIV-infected MSM in our study in Bogotá in 2010 were unaware of their serostatus prior to the HIV test we administered [2]. Increasing regular and repeated testing among sexually active MSM is crucial for the goal of lowering HIV incidence by decreasing untreated cases.

We found the online educational entertainment intervention to be acceptable to young MSM in Bogotá, Colombia. Participants commented that they felt they could relate to the story and the characters in the film, and that they found it enjoyable. More important, they felt this material fills a critical gap in the community. Our preliminary assessment of the intervention shows that watching the film increased knowledge of HIV rights. This effect was particularly strong for men who had never been tested at baseline. Knowledge of HIV transmission dynamics also increased, but it also increased in the control group. Thus, we cannot attribute this increase to the intervention.

Moderation analyses indicated that HIV testing intentions increased significantly more among non-gay-identified participants in the intervention group compared with gay-identified participants in the intervention and control groups. This finding is in line with that of Blas and colleagues, who found that the effect of their short intervention (5-min video) on intentions to get tested was significant only among non-gay-identified MSM in Peru [26]. Blas and colleagues argued that non-gay-identified MSM could be more susceptible to the impact of the intervention because they were less likely to have any HIV testing experience, while the intervention added little to gay-identified men, who were more experienced with testing. In our study, although lifetime HIV testing was high for all participants (75% reported ever testing at baseline), testing was slightly more common among gay-identified participants (81.8%) than among those who identified as bisexual, heterosexual or other (63.2%). Men who identify as gay are more likely to have gay social networks and to be exposed to information about sexual health than those who do not identify as part of the gay community [41]. Interventions promoting HIV testing among non-gay identified MSM are crucial and could help stem the epidemic. Our video intervention presented supportive parents, friends, and health professionals, and demystified HIV testing. It is possible that non-gay identified participants were influenced by the sexual orientation affirming nature of the video, as well as by the health messages it delivered but that gay identified participants did not need messages that many of them already endorse and have internalized; alternatively, they could be desensitized to these messages. We did not find a significant main effect of the intervention on HIV testing intentions.

Lastly, HIV testing uptake did not change from baseline to follow-up, and moderation effects were not significant either. The limited impact of the intervention on HIV testing uptake might be due to celling effects: contrary to our results in 2010, which suggested that only 50% of young MSM in Bogota had been tested for HIV in their lifetime, in 2017 most young MSM reported having been tested in the past and having high intentions to test in the future. This might be the result of multiple recent interventions in Colombia, such as the United Nations Population Fund’s campaigns to provide HIV testing among MSM and other populations in the largest cities in Colombia. Additional research with samples in smaller cities, where HIV testing is not offered as regularly and where supportive gay social networks and health professionals are scarce, is needed. This video intervention can supplement the gap of very much needed affirming messages in small cities. A more granular understanding of the way that decreasing stigma related to sexual orientation, HIV status, and HIV testing can promote health behavior should be part of future research agendas.

More research to understand why increases in knowledge and, among non-gay-identified MSM, increased HIV testing intentions did not result in changes in behavior is also needed. Diaz and Ayala identified early in the epidemic a disconnect between high levels of knowledge about transmission dynamics and sexual practices [42], and in this study we found a similar disconnect between knowledge of transmission dynamics and HIV testing uptake. Garcia et al. [11] and Parker et al. [43] argue that community based efforts to increase preventive behaviors remain insufficient and that we need a better understanding of how those most at risk for HIV understand, recognize and manage HIV risk in the contexts of their daily lives. We continue to believe that community-based participatory research is crucial, but also that we need to understand how knowledge can be translated into action by disentangling the mechanisms through which HIV-testing uptake can be increased.

It is important to recognize that some characteristics of the intervention might have affected its effectiveness. First, “*Bondage: It is better to know*” was an 83-min film, while other video-based interventions to promote HIV testing among MSM tend to be much shorter. For example, among the seven video-based eHealth interventions reviewed by Nguyen and colleagues [19], video length ranged between 1 min [22] and 20 min [25]. Previous research has found that intervention length can become a barrier to participant engagement [19,23]. Future studies could compare the impact of different edited versions of the film “*Bondage: It is better to know*” with varying lengths. Second, the film was used as a stand-alone intervention. While informative and entertaining, the film alone may not be sufficient to change intentions and behaviors. In their study about couples HIV testing and counselling as a platform to address drug use among male couples, Starks and colleagues found that a 20-min video alone was not effective in reducing drug use. When paired with a short motivational interviewing session, however, the video enhanced the long-term impact of the intervention, so that drug use at 3- and 6-months follow-ups decreased only for those who received both the video and the motivational interviewing session [25]. Adding materials and discussions could boost the effect of the intervention. Alternatively, the film could be added as a component of other interventions. A benefit of the video format is that it can be easily adapted for use in offline and online interventions, at the group or individual levels.

Our study has a number of limitations. First, although recruitment for this study was very successful, retention was more challenging. We recruited 300 participants for the baseline survey in less than a month, yet only 95 (31.7%) completed the follow-up survey. Frequent changes in cellphone numbers due to high theft in Bogotá could be responsible for our inability to retain baseline participants. Second, although participants were informed at the time of enrollment that the study could entail watching an online film or webnovela series, baseline participants’ response to our invitation to view the film was low. We invited 185 unknown HIV status or HIV-negative participants to view the film. Of 97 (52%) participants who opened the link, only 63 (34%) viewed the film and completed the post-viewing survey. It is possible that the lack of a financial incentive for viewing the film and completing the post-viewing survey affected participants’ willingness to devote time and effort [24]. It is also possible that study participants did not recall that the video was part of the study in which they had enrolled. Additionally, limited internet connectivity and data plans could have decreased participants’ ability to access the film. To overcome this barrier, organizations interested in this type of intervention could provide internet access. Other formats, such as group administration, could also be explored. Colombians are highly relational and young MSM may prefer group-based viewing. In instances in which people are in vulnerable situations (e.g., areas in which homonegativity is high) or in remote locations, group-based viewing may not be feasible, so private viewing becomes the only form of intervention delivery. The film allows the flexibility to use public and private viewing.

## 5. Conclusions

Using a CBPR approach, we developed and produced “*Bondage: It is better to know*”, an educational-entertainment film to promote HIV testing among young MSM in Bogotá, Colombia. To our knowledge, this is the first video-based intervention to be evaluated with Colombian MSM. Developing a film using CBPR approaches is feasible and the intervention was acceptable for the target population. Although preliminary evidence of effectiveness is limited, the intervention appears to promote knowledge of HIV-related rights, particularly among young MSM who have never been tested. Similarly, viewing the film was associated with increased HIV testing intentions among non-gay-identified participants. This study adds to our limited knowledge about the feasibility, acceptability and effectiveness of using video-based interventions to promote HIV testing among MSM in Latin America. The intervention is easily scalable and it could be used with flexibility to promote HIV testing among MSM in other cities and other Spanish-speaking countries, or among Latinos in the U.S.

## Figures and Tables

**Table 1:** Major activities, specific objectives, significant results and key outcomes for the first three steps regarding the development of the education-entertainment online intervention.

Major Activity	Specific Objectives	Significant Results/Key Outcomes
**Step 1: Background Research on the Target Population, the Message Topic, and the Communication Environment.**		
Three in-person CBPR team meetings.	To share our previous research findings in Colombia with the edu-entertainment team. To carry out new formative research on the target population and the message topic to inform the webnovela message. To find a common ground with CBPR regarding communication for social and behavioral change. To understand the communication environment in Colombia.	Formative research to inform the webnovela development and message.
Two focus groups with young MSM (n = 15).	To enrich formative research findings and assess current knowledge, attitudes, practices and social norms among young MSM regarding sexual life, HIV, HIV testing and communication.	Input for Message Brief.
**Step 2: Development of the Message Brief.**		
Message brief workshop (CBPR team and PI).	Based on formative research, to identify the crucial messages to bring about the desired changes in the target community.	Draft of Message Brief.
Two focus groups with young MSM (n = 15) & Key-informant interviews (N = 5)	Obtain feedback on the clarity and suitability of the Message Brief.	Feedback for Message Brief.
Message brief adjustment workshop (CBPR team and PI).	Examine feedback from focus groups and key-informant interviews, and revise the Message Brief accordingly.	Final Message Brief (See Figure 1).
**Step 3: Development of broad story outline and characters.**		
Recruitment of Creative team	Identify Producer, Director, Assistant Director and scriptwriters for film and production.	Creative team.
Creative workshop I (CBPR, PI, and Creative teams).	Discuss possible characters, setting, plot, and actions.	Findings were used for development of Draft Story Outline.
Creative team meetings.	Integrate the Message Brief into a drama, achieving the appropriate balance between entertainment and education.	Draft Story Outline.
Creative workshop II (CBPR, PI, and Creative teams).	Create the arc of the story and characters. Discuss Draft Story Outline and revise accordingly.	
Three focus groups with young MSM (n = 30)/Key informants interviews (N = 5).	Obtain feedback on the story, characters, and messages in the Draft Story Outline. Validation of the universe, treatment and summary of 13 chapters.	Feedback for Story Outline.
Creative workshop III (CBPR, PI, and Creative teams).	Examine feedback and determine aspects that need further changes.	Final Story Outline.
**Step 4: Development of the script.**		
Draft script (Creative team).	Based on final story outline, develop detailed story lines, character sketches, scripts, and scenes	Draft Script.
Three focus groups with young MSM (n = 51)/Key informants interviews (N = 5).	Obtain feedback on draft scripts	
Creative workshop III (CBPR, PI, and Creative teams).	Identify areas in need of modification and make recommendations to the creative team to revise the story lines, characters, and scripts.	Final Script.
**Step 5: Production.**		
Casting and hiring of production and direction teams	Pre-production	
Three focus groups with young MSM (n = 40)	Provide feedback on casting and final scripts.	
Filming and editing	Finalize product	Decision to use a feature film format, rather than a webnovela with 5 episodes. Final version of “Bondage It is better to know”.

**Table 2:** Baseline demographic characteristics of participants by group and time point.

Characteristic	Baseline Survey (N = 300) % (95% CI)	Post-Viewing Acceptability Survey (n = 63) % (95% CI)	Follow-Up Survey (n = 95)	
			Intervention Group (n = 48) % (95% CI)	Control Group (n = 47) % (95% CI)
Age (Mean ± SD)	23.18 ± 3.40	22.65 ± 3.32	22.29 ± 3.12	21.85 ± 3.01
Gender identity ^a^				
Cisgender men	95.0 (91.9–97.2)	98.4 (91.5–100)	100 (92.6–100)	93.6 (82.5–98.7)
Transgender women	3.7 (1.8–6.5)	1.6 (0.0–8.5)	0.0 (0.0–7.4)	4.3 (0.5–14.5)
Transgender men	1.3 (0.4–3.4)	0.0 (0.0–5.7)	0.0 (0.0–7.4)	2.1 (0.1–11.3)
Sexual orientation				
Gay	66.3 (60.7–71.7)	96.8 (57.0–80.8)	77.1 (62.7–88.0)	44.7 (30.2–59.9)
Bisexual	26.3 (21.4–31.7)	22.2 (12.7–34.5)	16.7 (7.5–30.2)	40.4 (26.4–55.7)
Heterosexual	4.3 (2.3–7.3)	7.9 (2.6–17.6)	6.3 (1.3–17.2)	12.8 (4.8–25.7)
Don’t know	3.0 (1.4–5.6)	0.0 (0.0–5.7)	0.0 (0.0–7.4)	2.1 (0.1–11.3)
Socio-economic strata ^b^				
Low	37.8 (32.1–43.8)	32.3 (20.9–45.3)	39.6 (25.8–54.7)	43.2 (28.3–59.0)
Medium	60.0 (53.9–65.8)	67.7 (54.7–79.1)	60.4 (45.3–74.2)	56.8 (41.0–71.7)
High	2.2 (0.8–4.7)	0.0 (0.0–5.8)	0.0 (0.0–7.4)	0.0 (0.0–8.0)
Education				
Less than high school	6.3 (3.9–9.7)	0.0 (0.0–5.7)	0.0 (0.0–7.4)	6.4 (1.3–17.5)
High school	20.7 (16.2–25.7)	15.9 (7.9–27.3)	16.7 (7.5–30.2)	21.3 (10.7–35.7)
Some college	52.7 (46.8–58.4)	63.5 (50.4–75.3)	60.4 (45.3–74.2)	57.4 (42.2–71.7)
College or more	20.3 (15.9–25.3)	20.6 (11.5–32.7)	22.9 (12.0–37.3)	14.9 (6.2–28.3)
Self-reported HIV status (At each time point)				
Positive	8.3 (5.5–12.1)	7.9 (2.6–17.6)	12.5 (4.7–25.2)	4.3 (0.5–14.5)
Negative	62.3 (56.6–67.8)	61.9 (48.8–73.9)	43.8 (29.5–58.8)	42.6 (28.3–57.8)
Unknown	29.3 (24.2–34.8)	30.2 (19.2–43.0)	43.8 (29.5–58.8)	53.2 (38.1–67.9)

Notes: ^a^ Baseline demographics stratified by gender identity are presented in Supplementary Materials Table S1. ^b^ Socio-economic strata in Colombia ranges from 1 (the poorest) to 6 (the wealthiest); we collapsed strata 1 and 2 (low), 3 and 4 (medium) and 5 and 6 (high).

**Table 3:** Acceptability of the education-entertainment intervention—Measures and Results (Post-viewing survey only; n = 63).

Scale/Item	Mean	SD	Range	α
How much did you enjoy *Bondage*?	3.79	1.02	1–5	
How informative did you find *Bondage*?	4.21	0.94	1–5	
How much do you think your MSM friends would enjoy the film *Bondage*?	3.81	1.13	1–5	
Would you recommend Bondage to a friend?	2.54	0.64	1–3	
*Narrative engagement*	3.47	0.73	1.3–4.9	0.83
Sometimes it was hard for me to understand what was going on in the film (Rev)	3.45	1.14	1–5	
Sometimes my mind wandered while I was watching the film (Rev)	3.37	1.28	1–5	
During the film, my body was in the room but my mind was in the world created by the story.	3.24	1.18	1–5	
The story affected me emotionally	2.81	1.16	1–5	
I felt sorry for some of the film characters	3.53	1.16	1–5	
I felt the whole time that I wanted to know how the story would end	3.92	1.01	1–5	
The story’s events are relevant for my daily life	3.18	1.17	1–5	
I could picture myself in the events shown in the film	3.48	1.24	1–5	
I reflected on the topics covered in the film	4.06	0.99	1–5	
I thought about the situations and motivations of the characters	3.68	1.08	1–5	
*Counterarguing*	2.62	0.96	1–4.75	0.76
I felt like criticizing my disagreement with what was happening or was being said	2.79	1.23	1–5	
I thought that the information on some topics was imprecise or wrong	1.90	1.20	1–5	
I considered different ways in which the story could have been different	3.23	1.31	1–5	
I tried to identify problems with the information around some topics	2.55	1.28	1–5	
*Identification with the main character (Gabriel)*	3.43	1.10	1–5	0.88
I felt as if I were GABRIEL	2.92	1.51	1–5	
I imagined how I would act if I were in GABRIEL’s place	3.79	1.37	1–5	
I worried about what was happening to GABRIEL	3.56	1.20	1–5	
I understood GABRIEL’s feelings or emotions	3.66	1.25	1–5	
I tried to see things from GABRIEL’s perspective	3.61	1.24	1–5	
I identified with GABRIEL	3.07	1.48	1–5	

**Table 4:** HIV testing behaviors and intentions, knowledge of HIV transmission dynamics and knowledge of HIV-related rights by group and time point.

Characteristic	Baseline Survey ^e^ (N = 300) Mean ± SD/ % (95% CI)	Post-Viewing Acceptability Survey (n = 63) Mean ± SD/ % (95% CI)	Follow-Up Survey (n = 95)	
			Intervention Group (n = 48) Mean ± SD/ % (95% CI)	Control Group (n = 47) Mean ± SD/ % (95% CI)
**HIV Testing ^a^ (% [95% CI])**				
Yes	74.7 (69.3–79.5)	79.3 (66.6–88.8)	56.3 (41.2–70.5)	46.8 (29.5–58.8)
No	25.3 (20.5–30.7)	20.7 (11.2–33.4)	43.8 (32.1–61.9)	53.2 (38.1–67.9)
HIV testing intentions ^b^ (Mean ± SD)	4.32 ± 0.89	4.19 ± 0.91	4.46 ± 0.61	4.41 ± 1.03
I plan to get an HIV test within the next 6 months	4.33 ± 0.97	4.20 ± 0.96	4.50 ± 0.67	4.42 ± 1.05
From now on, I plan to get tested for HIV on a regular basis	4.32 ± 0.95	4.18 ± 0.94	4.42 ± 0.63	4.40 ± 1.05
Knowledge about HIV transmission dynamics ^c^ (Mean ± SD)	2.45 ± 1.29	3.27 ± 1.26	3.40 ± 1.14	2.96 ± 1.32
There is a medication that, when taken properly, reduces HIV levels in blood and semen [True]	0.36 ± 0.48	0.58 ± 0.49	0.66 ± 0.47	0.51 ± 0.50
It is possible to know if a person is infected with HIV from how he/she looks (his/her appearance) [False]	0.54 ± 0.49	0.72 ± 0.44	0.66 ± 0.47	0.57 ± 0.49
A person can be infected with HIV and not have AIDS [True]	0.61 ± 0.48	0.83 ± 0.37	0.83 ± 0.37	0.70 ± 0.46
If a person has HIV but gets treatment on time he/she can live a normal life with a normal life span [True]	0.78 ± 0.41	0.91 ± 0.27	0.91 ± 0.27	0.87 ± 0.33
When a person living with HIV has an undetectable viral load, he can still transmit HIV to another person [False]	0.14 ± 0.35	0.20 ± 0.41	0.31 ± 0.46	0.29 ± 0.46
Knowledge about HIV-related rights ^d^ (Mean ± SD)	2.71 ± 1.06	3.11 ± 1.14	3.14 ± 0.87	2.85 ± 0.97
All Colombian citizens have the right to get treatment for HIV/AIDS [True]	0.80 ± 0.39	0.77 ± 0.42	0.79 ± 0.41	0.80 ± 0.39
Employers can demand their employees to get tested for HIV before hiring them [False]	0.37 ± 0.48	0.59 ± 0.49	0.62 ± 0.48	0.36 ± 0.48
HIV test results should be confidential [True]	0.84 ± 0.36	0.88 ± 0.31	0.95 ± 0.20	0.87 ± 0.33
By law, health-providing organizations must approve HIV tests, up to twice per year, to any member who requests it [True]	0.70 ± 0.45	0.85 ± 0.35	0.77 ± 0.42	0.80 ± 0.39

Notes: ^a^ HIV testing refers to lifetime for baseline, last 3 months for post-viewing and last 6-months for follow-up surveys. ^b^ Response scale for HIV testing intentions items ranged from 1 “Strongly disagree” to 5 “Strongly agree” (Range: 1–5). ^c^ Scored as the sum of items responded correctly (Range: 0–5). ^d^ Scored as the sum of items responded correctly (Range: 0–4). ^e^ Baseline characteristics stratified by gender identity are presented in Supplementary Materials Table S1.

**Table 5:** Means and standard deviations (SD) of HIV-related outcomes by group (intervention vs. control) and time-point (baseline vs. follow-up).

Outcome Variable	Time-Point	Group	
		Intervention (n = 48) Mean (SD)	Control (n = 47) Mean (SD)
Recent HIV testing ^a^	Baseline	0.48 (0.50)	0.43 (0.50)
	Follow-up	0.56 (0.50)	0.47 (0.50)
HIV testing intentions ^b^	Baseline	4.33 (0.93)	4.41 (0.65)
	Follow-up	4.46 (0.62)	4.41 (1.03)
Knowledge of HIV transm. Dynamics ^c^	Baseline	2.88 (1.25)	2.23 (1.17)
	Follow-up	3.40 (1.14)	2.96 (1.32)
Knowledge of HIV-related rights ^d^	Baseline	2.75 (1.16)	2.70 (0.86)
	Follow-up	3.15 (0.88)	2.85 (0.98)

Notes: ^a^ For baseline: Tested in the last 12 months; For follow up: Tested in the last 6 months; Response options: 0 = No–1 = Yes. ^b^ Response scale for HIV testing intentions items ranged from 1 “Strongly disagree” to 5 “Strongly agree” (Range: 1–5). ^c^ Scored as the sum of items responded correctly (Range: 0–5). ^d^ Scored as the sum of items responded correctly (Range: 0–4).

## Data Availability

The data presented in this study are available on request from the corresponding author. The data will be publicly available on December 2021.

## Supplementary Materials

The following are available online at https://www.mdpi.com/1660-4601/18/4/1811/s1, Table S1: Baseline characteristics by gender identity. Table S2: Measures—Spanish version.
