# Supplementary material for: Development and Evaluation of an Online Education-Entertainment Intervention to Increase Knowledge of HIV and Uptake of HIV Testing among Colombian Men Who Have Sex with Men (MSM)
Source: Int J Environ Res Public Health. 2021 Feb 12;18(4):1811. doi: 10.3390/ijerph18041811 (PMC7918496; doi:10.3390/ijerph18041811)
Supplement: Supplementary file 1 [file ijerph-18-01811-s001.zip › Supplemental Material 1 Baseline characteristics by gender identity.docx]

**Supplemental Material 1. Descriptive Statistics (Means and percentages) for Baseline Characteristics by Gender Identity.**

| **Characteristic** | **Total sample**  **(N = 300)** | **Cisgender men**  **(n = 285)** | **Transgender women (n = 11)** | **Transgender men (n = 4)** |
| --- | --- | --- | --- | --- |
|  | **% (95% CI)** | **% (95% CI)** | **% (95% CI)** | **% (95% CI)** |
| Sexual orientation |  |  |  |  |
| Gay | 66.3 (60.7 – 71.7) | 67.36 (61.58 - 72.78) | 45.45 (16.74 - 76.62) | 50.0 (6.76 - 93.24) |
| Bisexual | 26.3 (21.4 – 31.7) | 26.31 (21.29 - 31.83) | 18.18 (2.28 - 51.77) | 50.0 (6.76 - 93.24) |
| Heterosexual | 4.3 (2.3 – 7.3) | 3.86 (1.94 - 6.80) | 18.18 (2.28 - 51.77) | 0 |
| Don’t know | 3.0 (1.4 – 5.6) | 2.46 (0.99 - 4.99) | 18.18 (2.28 - 51.77) | 0 |
| Socio-economic strata^a^ |  |  |  |  |
| Low | 37.8 (32.1 – 43.8) | 37.12 (31.27 - 43.25) | 55.55 (21.20 - 86.30) | 50.00 (1.257 - 98.74) |
| Medium | 60.0 (53.9 – 65.8) | 60.60 (54.43 - 66.54) | 44.44 (13.69 - 78.79) | 50.00 (1.257 - 98.74) |
| High | 2.2 (0.8 – 4.7) | 2.30 (0.80 – 0.49) | 0 | 0 |
| Education |  |  |  |  |
| Less than high school | 6.3 (3.9 – 9.7) | 5.614 (3.24 - 8.96) | 27.27 (6.02 - 60.97) | 0 |
| High school | 20.7 (16.2 – 25.7) | 20.35 (15.83 - 25.49) | 18.18 (2.28 - 51.77) | 50.00 (6.76 - 93.24) |
| Some college | 52.7 (46.8 – 58.4) | 54.03 (48.05 - 59.92) | 36.36 (10.92 - 69.20) | 0 |
| College or more | 20.3 (15.9 – 25.3) | 20.00 (15.51 - 25.12) | 18.18 (2.28 - 51.77) | 50.00 (6.76 - 93.24) |
| Ever tested for HIV | 74.7 (69.3 – 79.5) | 75.1 (69.6 – 80.0) | 63.6 (30.8 - 89.1) | 75.0 (19.4 - 99.4) |
| Self-reported HIV status |  |  |  |  |
| Positive | 8.3 (5.5 – 12.1) | 8.42 (5.47 - 12.27) | 9.09 (0.23 - 41.27) | 0 |
| Negative | 62.3 (56.6 – 67.8) | 62.80 (56.91 - 68.43) | 45.45 (16.74 - 76.62) | 75.00 (19.41 - 99.36) |
| Unknown | 29.3 (24.2 – 34.8) | 28.77 (23.58 - 34.40) | 45.45 (16.74 - 76.62) | 25.00 (0.63 - 80.58) |
|  | **Mean ± SD** | **Mean ± SD** | **Mean ± SD** | **Mean ± SD** |
| Age | 23.18 ± 3.40 | 23.13 ± 3.33 | 24.64 ± 4.9 | 23 ± 2.94 |
| HIV testing intentions^b^ | 4.32 ± 0.89 | 4.32 ± 0.91 | 4.40 ± 0.66 | 4.50 ± 0.58 |
| I plan to get an HIV test within the next 6 months | 4.33 ± 0.97 | 4.33 ± 0.99 | 4.30 ± 0.95 | 4.50 ± 0.58 |
| From now on, I plan to get tested for HIV on a regular basis | 4.32 ± 0.95 | 4.31 ± 0.97 | 4.50 ± 0.71 | 4.50 ± 0.58 |
| Knowledge about HIV transmission dynamics^c^ | 2.45 ± 1.29 | 2.48 ± 1.30 | 2.18 ± 0.60 | 1.00 ± 0.82 |
| There is a medication that, when taken properly, reduces HIV levels in blood and semen [True] | 0.36 ± 0.48 | 0.36 ± 0.48 | 0.45 ± 0.52 | 0.25 ± 0.50 |
| It is possible to know if a person is infected with HIV from how he/she looks (his/her appearance) [False] | 0.54 ± 0.49 | 0.55 ± 0.50 | 0.55 ± 0.52 | 0.25 ± 0.50 |
| A person can be infected with HIV and not have AIDS [True] | 0.61 ± 0.48 | 0.63 ± 0.48 | 0.45 ± 0.52 | 0.00 ± 0.00 |
| If a person has HIV but gets treatment on time he/she can live a normal life with a normal life span [True] | 0.78 ± 0.41 | 0.79 ± 0.41 | 0.73 ± 0.47 | 0.50 ± 0.58 |
| When a person living with HIV has an undetectable viral load, he can still transmit HIV to another person [False] | 0.14 ± 0.35 | 0.15 ± 0.36 | 0.00 ± 0.00 | 0.00 ± 0.00 |
| Knowledge about HIV-related rights ^d^ | 2.71 ± 1.06 | 2.73 ± 1.06 | 2.73 ± 1.01 | 1.75 ± 1.26 |
| All Colombian citizens have the right to get treatment for HIV/AIDS [True] | 0.80 ± 0.39 | 0.81 ± 0.39 | 0.64 ± 0.5 | 0.75 ± 0.50 |
| Employers can demand their employees to get tested for HIV before hiring them [False] | 0.37 ± 0.48 | 0.37 ± 0.48 | 0.45 ± 0.52 | 0.00 ± 0.00 |
| HIV test results should be confidential [True] | 0.84 ± 0.36 | 0.84 ± 0.37 | 1.00 ± 0.00 | 0.75 ± 0.50 |
| By law, health-providing organizations must approve HIV tests, up to twice per year, to any member who requests it [True] | 0.70 ± 0.45 | 0.71 ± 0.46 | 0.64 ± 0.5 | 0.25 ± 0.50 |

Notes: a. Socio-economic strata in Colombia ranges from 1 (the poorest) to 6 (the wealthiest); we collapsed strata 1 and 2 (low), 3 and 4 (medium) and 5 and 6 (high). b. Response scale for HIV testing intentions items ranged from 1 “Strongly disagree” to 5 “Strongly agree” (Range: 1 – 5). c. Scored as the sum of items responded correctly (Range: 0 – 5). Mean values for the items represent the proportion of the sample that answered each item correctly. d. Scored as the sum of items responded correctly (Range: 0 – 4). Mean values for the items represent the proportion of the sample that answered each item correctly.
