# Supplementary material for: Development and Evaluation of an Online Education-Entertainment Intervention to Increase Knowledge of HIV and Uptake of HIV Testing among Colombian Men Who Have Sex with Men (MSM)
Source: Int J Environ Res Public Health. 2021 Feb 12;18(4):1811. doi: 10.3390/ijerph18041811 (PMC7918496; doi:10.3390/ijerph18041811)
Supplement: Supplementary file 1 [file ijerph-18-01811-s001.zip › Supplemental Material 2 Measures Spanish version.docx]

**Supplemental Material 2. Measures – Spanish version.**

| **Escala / ítem (Opciones de respuesta)** |
| --- |
| ¿Qué tanto le gustó Bondage? (1 “Nada” – 5 “Mucho”) |
| ¿Qué tan informativa le pareció Bondage? (1 “Nada” – 5 “Mucho”) |
| ¿Qué tanto cree usted que la película Bondage les gustaría a sus amigos hombres que tienen sexo con hombres? (1 “Nada” – 5 “Mucho”) |
| ¿Recomendaría usted Bondage a algún amigo? (1 “No”, 2 “Tal vez”, 3 “Sí”) |
| **Enganche narrativo** (1 “Totalmente en desacuerdo” – 5 “Totalmente de acuerdo”) |
| En algunos momentos, fue difícil para mí entender lo que estaba pasando en la película |
| A veces mi mente se perdía en otras cosas mientras veía la película |
| Durante la película, mi cuerpo estaba en el cuarto, pero mi mente estaba en el mundo creado por la historia |
| La historia me afectó emocionalmente |
| Sentí pesar por algunos personajes de la película |
| Todo el tiempo tenía deseos de saber cómo iba a terminar la historia |
| Los eventos en la historia son relevantes para mi vida cotidiana |
| Me podía imaginar a mí mismo en los eventos mostrados en la película |
| Reflexionaba sobre los temas que se trataban en la película |
| Pensaba sobre la situación y las motivaciones de los personajes |
| **Contra-argumentación** (1 “Nada” – 5 “Mucho”) |
| Sentía que quería criticar o mostrar mi desacuerdo con lo que estaba sucediendo o lo que se estaba diciendo |
| Pensaba que la información que se daba sobre algunos temas era inexacta o engañosa |
| Me puse a pensar sobre formas en las que la historia hubiera podido ser diferente |
| Intentaba identificar defectos en la información que se daba sobre algunos temas |
| **Identificación con el personaje principal (Gabriel)** (1 “Nada” – 5 “Mucho”) |
| Me he sentido como si yo fuera GABRIEL |
| He imaginado cómo actuaría yo si me encontrara en el lugar de GABRIEL |
| Me he sentido preocupado por lo que le sucedía a GABRIEL |
| He entendido los sentimientos o emociones de GABRIEL |
| He intentado ver las cosas desde el punto de vista de GABRIEL |
| Me he identificado con GABRIEL |
| **Intenciones de realizarse la prueba de VIH** (1 “Totalmente en desacuerdo” – 5 “Totalmente de acuerdo”) |
| Planeo hacerme la prueba de VIH en los siguientes 6 meses |
| De ahora en adelante, planeo hacerme la prueba de VIH regularmente |
| **Conocimiento sobre dinámicas de transmisión del VIH** (Verdadero o Falso) |
| Actualmente existe un medicamento que, cuando se toma correctamente, reduce el nivel de VIH de una persona en sangre y semen [Verdadero] |
| Es posible saber si una persona está infectada con el VIH por como se ve (por su apariencia) [Falso] |
| Una persona puede estar infectada con el VIH sin tener SIDA [Verdadero] |
| Si una persona con VIH recibe tratamiento a tiempo, puede tener una vida normal durante muchos años [Verdadero] |
| Cuando una persona que vive con el VIH tiene una carga viral indetectable, todavía puede transmitir el VIH a otra persona [Falso] |
| **Conocimiento sobre derechos relacionados con el VIH** (Verdadero o Falso) |
| Todo ciudadano colombiano tiene el derecho de recibir tratamiento para el VIH o el SIDA [Verdadero] |
| Los empleadores pueden exigir que sus empleados se hagan la prueba del VIH antes de contratarlos [Falso] |
| Los resultados de la prueba del VIH deben ser confidenciales [Verdadero] |
| Por ley, las Empresas Promotoras de Salud (EPS) deben aprobar la prueba de VIH, hasta dos veces por año, a cualquier afiliado que la solicite [Verdadero] |
